# Massive Thymic Hyperplasia in Graves Disease

**DOI:** 10.1210/jcemcr/luaf076

**Published:** 2025-05-08

**Authors:** Christopher Chan, Simon Ryder, Dianna Luong, Gaurav Puri

**Affiliations:** Logan Endocrine and Diabetes Service, Logan Hospital, Metro South Health, Meadowbrook, Brisbane, QLD 4131, Australia; School of Medicine and Dentistry, Griffith University, Southport, Brisbane, QLD 4222, Australia; Logan Endocrine and Diabetes Service, Logan Hospital, Metro South Health, Meadowbrook, Brisbane, QLD 4131, Australia; Logan Endocrine and Diabetes Service, Logan Hospital, Metro South Health, Meadowbrook, Brisbane, QLD 4131, Australia; School of Medicine, University of Queensland, Herston, Brisbane, QLD 4006, Australia; Logan Endocrine and Diabetes Service, Logan Hospital, Metro South Health, Meadowbrook, Brisbane, QLD 4131, Australia; Clinical Excellence Queensland, Herston, Brisbane, QLD 4006, Australia

**Keywords:** thymic hyperplasia, Graves disease, anterior mediastinal mass

## Abstract

Thymic hyperplasia (TH) is a rare but established complication of Graves disease (GD). It is a benign phenomenon that regresses with appropriate treatment of GD. We report the case of a Burmese refugee with newly diagnosed GD, who had an incidental thymic mass measuring 70 mm × 21 mm × 121 mm. The size of the mass exceeded typical reports in the literature but was not accompanied by a substantial thyrotoxicosis. As the mass receded with antithyroid medication, a presumptive diagnosis of TH was ultimately made. Recognition of this association is vital to prevent unnecessary biopsy and surgical intervention.

## Introduction

Graves disease (GD) is an autoimmune disease characterized by autoantibodies against the thyrotropin receptor (TSH-R), which causes thyroid gland hyperplasia and excess thyroid hormone synthesis [1]. It is the most common cause of hyperthyroidism and can result in substantial morbidity and mortality through congestive heart failure, thyroid eye disease, and osteoporosis. First described in 1914 [2], thymic hyperplasia (TH) arising from GD is a rare but well-established complication with at least 100 previous reports in the literature [1]. The pathophysiology of TH in GD is not entirely understood. Thyroid hormone has been shown to exert a trophic effect on thymic epithelial and lymphoid cells [1, 3]. Thyrotropin receptor antibodies (TRAbs) may also stimulate extrathyroidal TSH-R, which has been demonstrated to exist on the thymus [4]. Notably, detection of a discrete mass, invasiveness, and calcification on imaging should prompt biopsy or further imaging as the differential diagnosis of thymic enlargement includes lymphoma, germ cell and mesenchymal tumors, thyroid and parathyroid masses, and metastatic malignancy. However, it is well established that TH regresses with adequate treatment of GD, typically with antithyroid drugs (ATDs). Thus, in the absence of concerning imaging findings, the literature supports medical management of an anterior mediastinal mass (AMM) in GD in the first instance [1].

We report a case of a Burmese refugee with newly diagnosed GD who had an incidental massive thymic mass identified on imaging that is among the largest reported in the literature. While there was no histological diagnosis, resolution of the mass with treatment of GD led to a presumptive diagnosis of TH.

## Case Presentation

A 41-year-old female Burmese refugee presented to our emergency department with 2 weeks of palpitations, tremors, diarrhea, and fatigue following arrival to Australia. Additionally, she reported the onset of a frontal headache, nausea, and vomiting for the previous 2 days, as well as 10 kg of unintentional weight-loss over the past month. Six weeks prior to admission, she had been newly diagnosed with thyrotoxicosis and diabetes mellitus by a general practitioner in Thailand and commenced on methimazole 15 mg daily, propranolol 10 mg twice daily, and metformin 500 mg daily. She denied other past medical history or family history.

## Diagnostic Assessment

On examination, there was a fine peripheral tremor of the upper limbs and a diffuse goiter with no discrete nodules. Hepatomegaly was noted without splenomegaly or cervical lymphadenopathy. Pemberton sign was positive. There was no evidence of thyroid eye disease, thyroid dermopathy, or congestive heart failure. She was initially bradycardic to 35 beats per minute, which resolved following reduction in propranolol dose to 5 mg. Initial telemetry revealed evidence of bigeminy with spontaneous reversion.

Initial thyroid function tests (TFTs) in Thailand, prior to the commencement of methimazole, returned a free thyroxine level (FT4) of 3.19 ng/dL (0.89-1.76 ng/dL) (41.1 pmol/L [11.5-22.7 pmol/L]) and a thyrotropin (TSH) level of less than 0.01 μIU/mL (0.55-4.78 μIU/mL) (<0.01 mIU/L [0.55-4.78 mIU/L]). Repeat TFTs at the time of admission to our service demonstrated improvement, with FT4 1.32 ng/dL (17.0 pmol/L), TSH less than 0.01 μIU/mL (<0.01 mIU/L), free 3,5,3′-triiodothyronine (FT3) 4.43 pg/mL (6.8 pmol/L), and TRAbs 6.5 IU/L (<1.8 IU/L).

**Figure 1.**
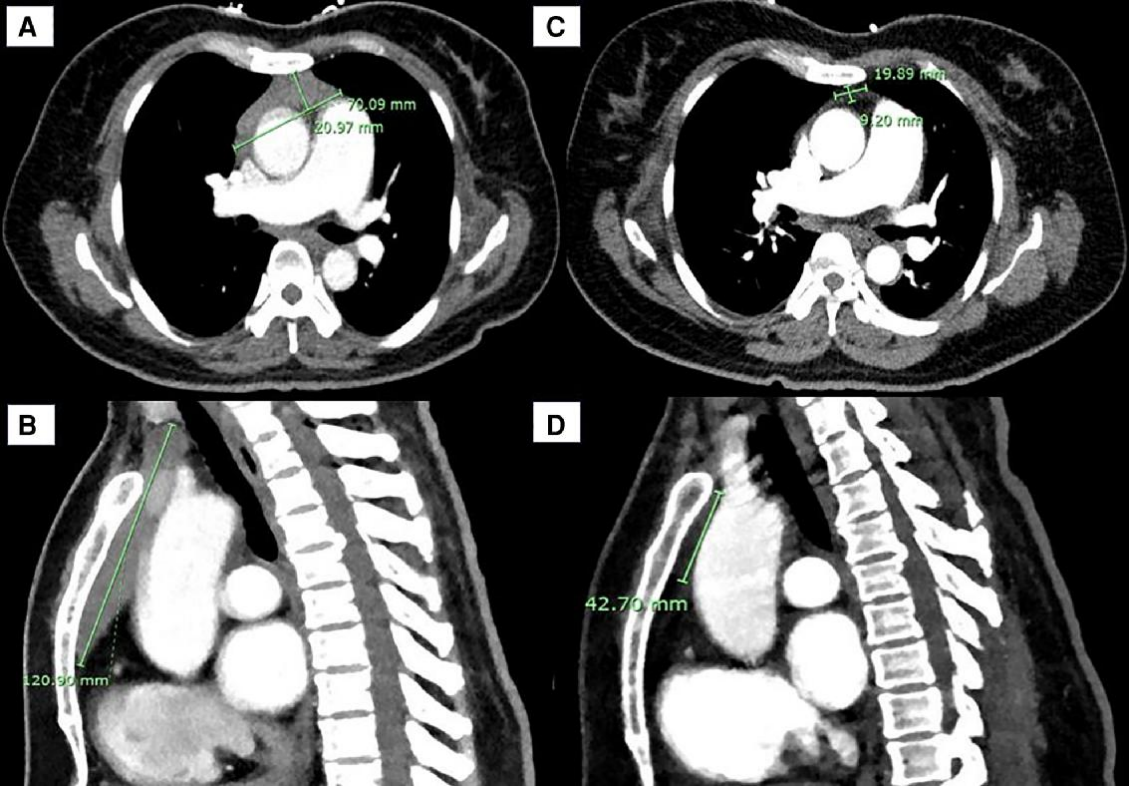
Anterior mediastinal mass (AMM) on computed tomography (CT) of the chest in A, transverse and B, sagittal views on admission to our service, 1 month following onset of Graves disease treatment. Interval CT chest in C, transverse and D, sagittal views 1 year after initial imaging, demonstrating regression of the thymic mass.

Chest x-ray was significant for an enlarged cardiac and mediastinal contour with a prominent aortic knuckle. A computed tomography (CT) of the head/angiogram, requested for vertiginous symptoms, revealed an incidental AMM. Follow-up CT chest with contrast showed a 70-mm × 21-mm × 121-mm (178 cm^3^) soft tissue–attenuating lesion with radiological reporting favoring thymic hyperplasia (Fig. 1). The massive volume of the mass exceeded typical volumes for TH described in the literature. Thus, the patient was broadly investigated for autoimmune conditions and malignancy. The mass demonstrated mild fluorodeoxyglucose (FDG) avidity on FDG positron emission tomography/CT, with minimal, nonspecific activity in bilateral cervical, axillary, periportal and femoral lymph nodes. Magnetic resonance imaging (MRI) of the mass did not demonstrate invasive features and favored TH with a chemical shift ratio of 0.6.

## Treatment

CT-guided biopsy was initially considered given the presence of lymph node FDG avidity. However, given that imaging demonstrated clear regression and no concerning features, and following extensive multidisciplinary discussions, the patient was treated conservatively with continuation of ATDs.

## Outcome and Follow-up

Interval CT of the chest 3 months following the patient’s initial imaging demonstrated considerable reduction in the size of the mass, with concave margins and internal fat density in keeping with TH. There was no evidence of lymphadenopathy or metastatic lesions. Similarly, the thymus measured 14 mm × 21 mm on her interval MR of the chest, representing a regression of 86% compared to 5 months previously. At 1 year following initial imaging, CT chest demonstrated normal size and morphology of the thymus (see Fig. 1).

She was followed-up by cardiothoracic surgery, who subsequently discharged her given resolution of the mass with ATDs. She was reviewed by neuroimmunology, who excluded myasthenia gravis in the absence of fatigable weakness and normal serology. She was converted from methimazole to carbimazole due to local drug availability, and was progressively weaned to 5 mg. Her most recent TFTs demonstrate euthyroidism: TSH 0.6 μIU/mL (0.6 mIU/L), FT4 0.95 ng/dL (12.3 pmol/L), FT3 2.47 pg/mL (3.8 pmol/L), and TRAbs 0.76 IU/L. At the time of writing, her propranolol has been discontinued, though she remains on 5 mg daily carbimazole with a plan for next follow-up in 3 months.

## Discussion

Detection of an AMM inevitably raises concern for malignancy and may lead to biopsy or surgical resection. TH is a well-established cause of AMM in GD, though the incidence is difficult to characterize as it often an incidental imaging finding [1]. While TH is often asymptomatic, it may present with pain, neck fullness, chest discomfort, and shortness of breath. In this case, the patient's positive Pemberton sign may be explained by the combination of her goiter and massive TH. Thymic size secondary to TH varies widely in the literature and is inconsistently reported as area or volume. In a study of 13 patients with GD and associated TH, the mean thymic size was reported as 852 mm^2^ (751 mm^2^ in 5 patients aged 40-49 years) prior to treatment [4]. TH measuring 226 cm^3^ on CT imaging has previously been reported [5]. Notably, TH is a benign phenomenon that resolves with adequate treatment of GD. In a review of 21 case reports of AMM in GD, all cases that were treated conservatively regressed with ATDs [6]. Regression is expected after 6 months of treatment and has been reported between 33% to 90% [1, 6]. Thymectomy is not without risk, with previous reports of phrenic nerve paralysis during investigation of an AMM in GD [6].

While there are currently no clinical guidelines that inform the diagnostic work-up or management of AMM in GD, the literature supports treatment of underlying GD in the first instance if initial CT of the chest demonstrates a homogenous mass without invasive features or calcification. Failure of the mass to regress on 6-month interval imaging is not characteristic of TH and warrants further investigation for alternative diagnoses including lymphoma, germ cell tumors, neuroendocrine tumors, paraganglioma, and other primary neoplasms. In the appropriate clinical context, investigation with tumor markers, flow cytometry, metanephrines, and hormone assays can thus be considered. Due to the association between myasthenia gravis and thymoma, detection of an AMM should prompt assessment for fatigable weakness and screening for acetylcholine receptor and muscle-specific kinase antibodies [1]. Chemical-shift MRI has been suggested as a useful, noninvasive, diagnostic tool in the work-up of AMM in GD [7]. In TH, chemical-shift MRI reveals a relative signal loss owing to fatty infiltration of the thymus gland. In contrast, there is no significant signal change detected in neoplastic processes that lack adipose tissue such as lymphoma [7]. Given the substantial size of the AMM in this case, which exceeded typical sizes reported in the literature, a broad differential diagnosis was considered (Table 1). However, with interval imaging demonstrating regression of the mass with ATDs, a presumptive diagnosis of TH was made, and biopsy was avoided. Furthermore, chemical-shift MRI demonstrated a signal loss that was supportive of the working diagnosis of TH.

**Table 1.** Investigations performed in our patient during work-up of their anterior mediastinal mass

Test	Result	Reference range
LDH	130 unit/L (2.2 µkat/L)	120-250 unit/L (2.0-4.2 µkat/L)
AFP	<0.4 ng/mL (<0.4 µg/L)	<10 ng/mL (<10 µg/L)
Free β hCG	0.08 mIU/mL (0.08 IU/L)	0.01-0.23 mIU/mL (0.01-0.23 IU/L)
Total hCG	0.3 mIU/mL (0.3 IU/L)	0.1-6 mIU/mL (0.1-6 IU/L)
Plasma 3-Methoxytyramine	6.5 pg/mL (39 pmol/L)	<15.1 pg/mL (<90 pmol/L)
Plasma metadrenaline	25.1 pg/mL (127 pmol/L)	5.9-88.8 pg/mL (30-450 pmol/L)
Plasma normetadrenaline	47.8 pg/mL (261 pmol/L)	22.0-146.5 pg/mL (120-800 pmol/L)
Urine 5-HIAA	2.5 mg/24 h (13 µmol/24 h)	7.6 mg/24 h (<40 µmol/24 h)
Chromogranin A	41 ng/mL (41 µg/L)	20-102 ng/mL (20-102 µg/L)
Flow cytometry	No abnormal clonal cell B-cell population	NA
ACHR Ab	Negative	NA
MUSK Ab	Negative	NA

Values are provided in conventional units (CU). Values in parenthesis are international system of units (SI). Plasma metanephrines were collected when patient was supine and fasted.

Abbreviations: 5-HIAA, 5-hydroxyindoleacetic acid; ACHR Ab, acetylcholine receptor antibody, AFP, α-fetoprotein; hCG, human chorionic gonadotropin; LDH, lactate dehydrogenase; MUSK Ab, muscle-specific kinase antibody; NA, not available.

TH encompasses true thymic hyperplasia or thymic lymphoid hyperplasia (TLH), which can only be differentiated based on histology [1]. True thymic hyperplasia is characterized by microscopic parenchymal hyperplasia of the thymic medulla and cortex. It is not commonly associated with autoimmune disease and is more prevalent in children as well as young men [8]. Comparatively, there is increased formation of medullary lymphoid follicles in TLH [1]. TLH is more commonly associated with autoimmune diseases such as myasthenia gravis and GD [9]. Thus, detection of a thymic mass warrants screening for these autoimmune conditions. Two primary mechanisms for TH in GD have been proposed in the literature, First, it has been demonstrated that thyroid hormone induces the growth of epithelial and lymphoid cells of the thymus. This action occurs primarily at the thymic cortex, and to a lesser extent the thymic medulla [1]. The exact mechanism by which thyroid hormone exerts this effect is unclear but may involve thyroid hormone modulation of thymulin, a thymic factor [3]. Second, expression of extrathyroidal TSH-R has been detected in the thymus [4]. In this setting, TRAbs may directly stimulate growth of the thymus, perhaps in a comparable fashion to thyroid eye disease and dermopathy [10].

Interestingly, there does not appear to be a dose-dependent relationship between the severity of thyrotoxicosis and extent of TH. A retrospective study of 40 patients with GD and associated TH demonstrated no statistically significant correlation between thymic volume, T4, T3, or TRAbs [11]. Indeed, the massive size of the thymic mass in this case was seemingly disproportionate to the degree of thyrotoxicosis.

This case was limited by the absence of chest imaging and TRAb levels prior to commencement of ATDs. This would have improved the comparison of pretreatment and posttreatment thymic volumes. Further, as with most cases of AMM in GD, the diagnosis of TH was presumed in the absence of a histological diagnosis.

## Learning Points

- Detection of an anterior mediastinal mass in GD should prompt consideration of TH.
- In the absence of concerning features on imaging, a conservative approach with ATDs in the first instance often leads to regression of the mass and a presumptive diagnosis of TH.
- Chemical-shift MRI is a valuable, noninvasive imaging modality that assists with differentiation between malignant processes and TH.
- There does not appear to be a dose-dependent relationship between TRAbs, T4, T3, and thymic volume.

## Contributors

All authors made individual contributions to authorship. C.C. was involved in the creation of the manuscript. S.R., D.L., and G.P. were involved in the management of the patient and provided feedback on the manuscript. All authors reviewed and approved the final draft.

## Data Availability

Data sharing is not applicable to this article as no data sets were generated or analyzed during the present study.

## Abbreviations

AMM: anterior mediastinal mass
ATDs: antithyroid drugs
CT: computed tomography
FDG: fluorodeoxyglucose
FT3: free 3,5,3′-triiodothyronine
FT4: free thyroxine
GD: Graves disease
MRI: magnetic resonance imaging
TFTs: thyroid function tests
TH: thymic hyperplasia
TLH: thymic lymphoid hyperplasia
TRAbs: thyrotropin receptor antibodies
TSH: thyrotropin
TSH-R: thyrotropin receptor
